# Microstructural, Fluid Dynamic, and Mechanical Characterization of Zinc Oxide and Magnesium Chloride-Modified Hydrogel Scaffolds

**DOI:** 10.1021/acsbiomaterials.4c00286

**Published:** 2024-07-16

**Authors:** Murilo Daniel de Mello Innocentini, Bruno Ribeiro Fuzatto Bueno, Agnieszka Urbaś, Anna Morawska-Chochół

**Affiliations:** † Course of Chemical Engineering, University of Ribeirão Preto, Avenida Costabile Romano 2201, 14096-900 Ribeirão Preto, SP, Brazil; ‡ Department of Architecture and Civil Engineering, Centre for Regenerative Design and Engineering for a Net Positive World (RENEW), University of Bath, Bath BA2 7AY, U.K.; § Faculty of Electrical Engineering, Automatics, Computer Science and Biomedical Engineering, AGH University of Krakow, 30-059 Kraków, Poland; ∥ Faculty of Materials Science and Ceramics, Department of Biomaterials and Composites, AGH University of Krakow, 30-059 Kraków, Poland

**Keywords:** composite scaffolds, zinc oxide, magnesium, nanohydroxyapatite, permeability, biomimetic

## Abstract

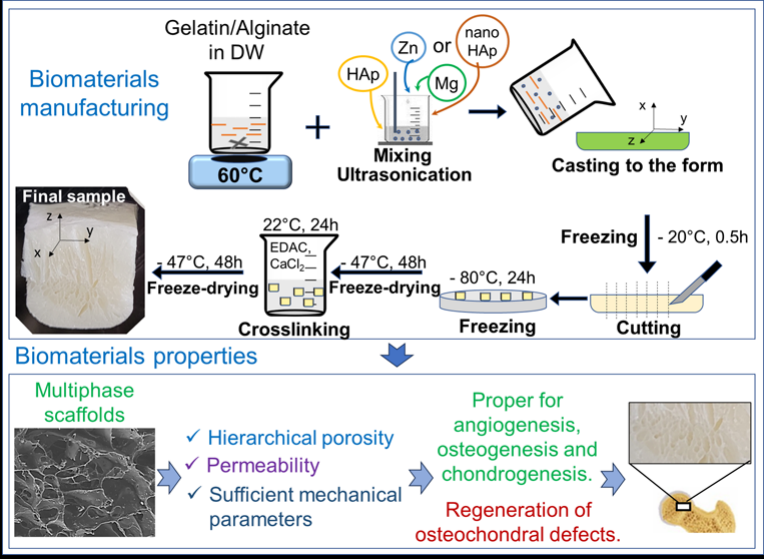

Scaffolds for the filling and regeneration of osteochondral defects are a current challenge in the biomaterials field, and solutions with greater functionality are still being sought. The novel approach of this work was to obtain scaffolds with biologically active additives possessing microstructural, permeability, and mechanical properties, mimicking the complexity of natural cartilage. Four types of scaffolds with a gelatin/alginate matrix modified with hydroxyapatite were obtained, and the relationship between the modifiers and substrate properties was evaluated. They differed in the type of second modifier used, which was hydrated MgCl_2_ in two proportions, ZnO, and nanohydroxyapatite. The samples were obtained by freeze-drying by using two-stage freezing. Based on microstructural observations combined with X-ray microanalysis, the microstructure of the samples and the elemental content were assessed. Permeability and mechanical tests were also performed. The scaffolds exhibited a network of interconnected pores and complex microarchitecture, with lower porosity at the surface (15 ± 7 to 29 ± 6%) and higher porosity at the center (67 ± 8 to 75 ± 8%). The additives had varying effects on the pore sizes and permeabilities of the samples. ZnO yielded the most permeable scaffolds (5.92 × 10^–11^ m^2^), whereas nanohydroxyapatite yielded the scaffold with the lowest permeability (1.18 × 10^–11^ m^2^), values within the range reported for trabecular bone. The magnesium content had no statistically significant effect on the permeability. The best mechanical parameters were obtained for ZnO samples and those containing hydrated MgCl_2_. The scaffold’s properties meet the criteria for filling osteochondral defects. The developed scaffolds follow a biomimetic approach in terms of hierarchical microarchitecture and mechanical parameters as well as chemical composition. The obtained composite materials have the potential as biomimetic scaffolds for the regeneration of osteochondral defects.

## 1 Introduction

Hydrogels are an extremely interesting group of materials in terms of their applicability as scaffolds for tissue engineering.^1^ This group of materials includes gelatin and calcium alginate. Gelatin is a natural biopolymer that results from collagen hydrolysis. This hydrogel, as a natural protein, has high bioaffinity, which promotes tissue regeneration. Alginate is a polysaccharide that is in high demand due to its widespread availability, low cost, and ease of cross-linking and drug incorporation. These materials are characterized primarily by similarity to the structure of the extracellular matrix, biocompatibility, high swelling capacity, and degradation in the biological environment. They also present advantages in processing and low production costs as well as the ability to control the properties by the degree of cross-linking.

Scaffolds for tissue engineering should meet a number of microstructural, mechanical, chemical, and biological requirements.^2^ Individual biological tissues differ in porosity, cell size, degree of vascularization, innervation, and the presence of lymphatic vessels, as well as strength and elasticity. All of these features affect the regenerative potential of biological structures. Thus, scaffolds intended for regeneration should be designed by considering the specifics of the selected tissue type. The best solution so far is the biomimetic approach, which consists of mapping the microstructure and microarchitecture of natural tissue. The most important microstructural requirements include the appropriate porosity and pore size, which depend on the type of tissue for which the scaffold is intended. These parameters should be selected in such a way as to allow for specific cells to migrate inside the scaffold. In addition, a key parameter is the permeability of the scaffolds, which testifies to the presence of the interconnected pores.^3^

The microarchitecture of pores, i.e., their shape, geometry, surface development, and interconnection, affects the permeability of such scaffolds for physiological fluids and the possibility of proliferation and migration of the selected cell type (cell’s permeability).^4^

Cartilage tissue is characterized by a low regenerative potential, which is due to the lack of innervation and vascularization and the lack of metabolically active cells. Cartilage regeneration techniques are based on drilling and microfractures, aimed at exposing the subchondral layer and bone, which is vascularized and has great regenerative potential. The scaffold intended for the regeneration of osteocartilage defects should, therefore, show a gradient of the microstructure or a layered structure that will reflect the structure of articular cartilage.^5^ Gradient characteristics of osteochondral tissue concern biochemical composition, microstructure, and mechanical properties.^6^ In cartilage tissue, 3 zones can be distinguished: cartilage (noncalcified), calcified cartilage, and subchondral bone. Noncalcified cartilage is characterized by a porosity of 60–85% and interconnected pores with a size of 2–6 nm. The compressive modulus of cartilage changes from the superficial to the deep zone from 0.2 to 6.44 MPa, and compressive strength changes from 0.005 to 4 MPa.^6,7^ Calcified cartilage is located between the cartilage and subchondral bone. The pore size and porosity of this zone as well as the compressive modulus are gradually varied. Chondrocyte number, size, and morphology are also different in this zone. Subchondral bone contains both cortical and trabecular bone; therefore, the porosity varies from 5 to 90%, and the pore size changes from 0.1 to 2000 μm.^6^ Gradient and anisotropy of subchondral bone affect the gradient and anisotropy of mechanical parameters of this zone. The elastic modulus of cortical bone is 14–22 GPa in the longitudinal direction and approximately 10 GPa in the transverse direction. Elastic modulus of trabecular bone is 0.1–0.9 GPa.^6,7^ The compressive strength of cortical bone is 188–222 MPa in the longitudinal direction and 110–150 MPa in the traverse direction. Compressive strength of trabecular bone ranges from 1 to 10 MPa.^6,7^ Subchondral bone contains different types of cells, such as osteoblasts, osteoclasts, osteocytes, and mesenchymal stem cells and, therefore, has great regenerative potential. Subchondral bone is built from mineral (hydroxyapatite) and organic (collagen) materials.^6^

Due to the complexity of osteochondral tissue, scaffolds designed for its regeneration must meet a number of parameters. Considering the microstructure, migration of the revenant cells should be possible into the pores; therefore, the scaffold pore size should be larger than the dimension of the cells. For optimum bone regeneration, scaffolds with porosity greater than 50% and pores larger than 300 μm are required.^8^ However, the smaller pores (at least 40 μm) permit the interchange of metabolic components and the adhesion of the cells. Due to these gradient factors, the bone scaffold should facilitate angiogenesis, bone cell migration, and the movement of physiological fluids (exchanging nutrients, oxygen, and metabolic waste). As a result, osteogenesis and vascularization become possible. The pore size of scaffolds for cartilage regeneration should be 90–120 μm, which is favorable for MSC proliferation and chondrogenesis.^9^ The other side of the scaffold should permit chondrocyte proliferation and feature pores with a somewhat smaller diameter. As demonstrated in the literature, such a gradient structure is more advantageous for the regeneration of osteochondral tissue.^6^

Magnesium plays a key role in the growth and development of the skeleton; it has been found that a magnesium deficiency can lead to cartilage damage. Additionally, magnesium ions play a role in mesenchymal stem cell (MSC) proliferation and chondrogenesis, and low doses may promote proliferation and high cell differentiation.^10−13^ Magnesium is essential for the interaction of the MSC with the extracellular matrix. Magnesium has been shown to increase MSC adhesion, promoting the formation of a cartilaginous matrix as well as increasing adhesion to collagen. Magnesium has a beneficial effect on the proliferation and redifferentiation of chondrocytes. Magnesium compounds also have a beneficial effect on osteogenesis.^11^ The combination of the bioactive action of hydroxyapatite (HAp) with magnesium ions seems to be an interesting solution that was taken up in the presented work. Hydroxyapatite as a natural bone component is characterized by biocompatibility, osteoconductivity, and bioactivity. HAp is a well-known and popular material used to support bone regenerative processes.^14,15^

Zinc oxide (ZnO) is a nontoxic compound characterized by biocompatibility and antibacterial activity for drug-resistant microbes.^16−18^ Due to the risk of perioperative bacterial infections associated with surgical intervention, giving the scaffolds antimicrobial properties is an interesting challenge. Zinc oxide appears to be a promising anti-infection modifier.

Ceramic additives have a significant impact on the microarchitecture and mechanical properties of scaffolds.^19−21^ Their impact on scaffold properties is inextricably linked to the type, content, and size of particles as well as the type of matrix–additives interfaces, which varies depending on polymer type. As a result, the role of modifiers in the literature varies. Another critical issue is the relationship between high porosity, gradient pore distribution, and scaffold mechanical properties. The additives not only strengthen the scaffolds but also reduce their porosity and pore size, which may have an effect on permeability.^19−21^ As a result, the effect of modifiers on these parameters must be understood.

The purpose of this research was to develop biomimetic composite scaffolds with a gradient structure from a gelatin–alginate matrix to regenerate bone cartilage defects. These hydrogels were chosen for their high biocompatibility and versatile processing capabilities. The combination of alginate and gelatin opens up new possibilities for shaping properties, particularly mechanical parameters, thanks to interpenetrating polymer networks.^1^ Zinc oxide, magnesium chloride, and hydroxyapatite were used to modify the scaffolds. The additives used are intended to stimulate regenerative processes while also providing antimicrobial activity. The paper investigates the effect of the additive type and its contribution to the mechanical parameters, microstructure, and permeability of the scaffolds.

## 2 Material and Methods

### 2.1 Materials

The following reagents were used in the fabrication of scaffolds: gelatin (CAS 9000-70-8, Poch); alginic acid (CAS 9005-38-3, Acros Organics); hydroxyapatite powder, HAp (CAS 1306-06-5, Acros Organics); hydroxyapatite nanopowder, nHAp (size 99% <100 nm CAS 1306-06-5, n-Gimat); magnesium chloride hexahydrate, MgCl_2_·6H_2_O (CAS 7791-18-6, Poch); zinc oxide, ZnO (CAS 1314-13-2, Macron Fine Chemicals); phosphate-buffered saline, PBS (Sigma-Aldrich); calcium chloride, CaCl_2_ (CAS 10043-52-4, Poch); and *N*-(3-(Dimethylamino)propyl)-*N*′-ethylcarbodiimide hydrochloride, EDAC (CAS 25952-53-8, Sigma-Aldrich).

### 2.2 Sample Preparation

Four types of hydrogel scaffolds with the following weight fraction of additives were obtained, as described in Table 1.

**Table 1:** Hydrogel Compositions Prepared In This Work (the Content of Additives Is Expressed as a Percentage by Weight Related to the Dry Mass of Hydrogel)

code	composition
GA_6H_4Mg	gelatin/alginate + 6% HAp + 1.90% MgCl_2_
GA_6H_6Mg	gelatin/alginate + 6% HAp + 2.88% MgCl_2_
GA_6H_4Mg_1nH	gelatin/alginate + 6% HAp + 1.90% MgCl_2_ + 1% nHAp
GA_6H_4Zn	gelatin/alginate + 6% HAp + 4% ZnO

**Figure 1:**
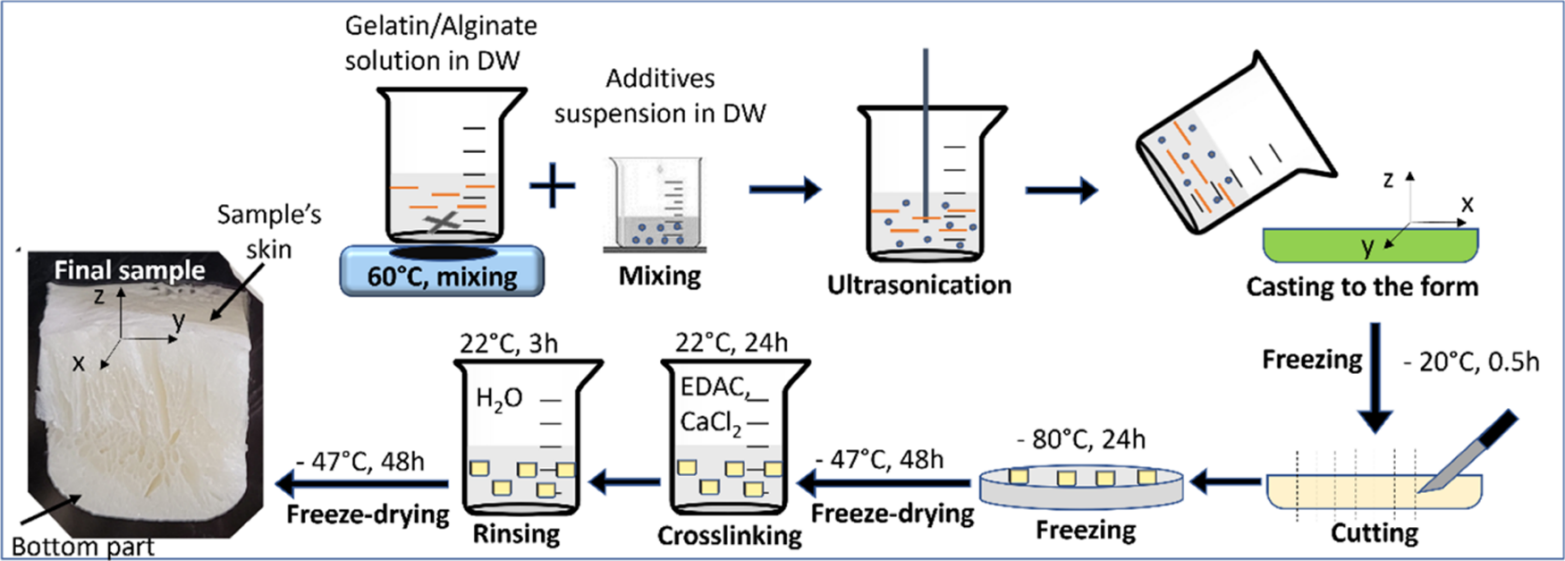
Scheme of sample preparation and the final sample.

The solution of gelatin and alginate was prepared by dissolving the respective powders in distilled water in a mass proportion of 4:1 (1.6 g of gelatin and 0.4 g of sodium alginate in 25 mL of distilled water). The HAp and ZnO powders were suspended in 5 mL of distilled water and then combined with a hydrogel solution. Similarly, 5 mL suspensions of HAp or HAp with nHAp in distilled water were prepared. Next, the MgCl_2_·6H_2_O modifier was dissolved in appropriate suspension and then combined with a hydrogel solution. The masses of additives were 127.66 mg of HAp (every sample); 0.20 mg of nano-HAp (GA_6H_4Mg_1nH); 83.33 mg of MgCl_2_·6H_2_O (GA_6H_4Mg); 127.66 mg of MgCl_2_·6H_2_O (GA_6H_6Mg), and 83.33 mg of ZnO (GA_6H_4Zn). The weight percentages of the additives (related to the dry mass of polymer) were 6 wt % of HAp, 1 wt % of nano-HAp, and 4 or 6 wt % of MgCl_2_·6H_2_O, which correspond, respectively, to 1.90 and 2.88 wt % of MgCl_2_ (the content of Mg was 0.49 and 0.74 wt %, respectively, related to the dry mass of polymer) and 4 wt % of ZnO (the content of Zn was 3.34 wt % related to the dry mass of polymer). The contents of additives were selected with regard to the maximum doses of metals safe for cells.^12,13^ The solution with additives was ultrasonicated for 30 s at an amplitude of 40%, poured into the 25 mL mold, and frozen at −20 °C for 0.5 h. Next, the samples were cut into pieces (≈14 mm × 14 mm × 12 mm) and frozen at −80 °C by 24 h (ULTF80 Arctico freezer). In the next step, the samples were freeze-dried for 48 h at 0.08 mbar and −47 °C (LABCONCO freeze-dryer). After that, the samples were cross linked in the solution of EDAC and CaCl_2_ (1 and 0.5 wt %, respectively) by 24 h. Next, the scaffolds were rinsed in distilled water for 3 h, frozen at −80 °C for 24 h (ULTF80 Arctico freezer), and lyophilized again at the same parameters as previously described. The particular steps of samples manufacturing are presented in Figure 1. Pure gelatin/alginate scaffold was obtained for the mechanical tests and was marked as GA.

### 2.3 Microstructural Evaluation

The microstructure of scaffolds was assessed using an optical digital microscope Keyence VHX-900F. Microscopic observations were also made using an ultrahigh-resolution scanning electron microscope (SEM) with a field-emission beam (FEG—SCHOTKYE emitter)—NOVA NANO SEM 200 (manufacturer of FEI EUROPE COMPANY) cooperating with the EDAX EDS analyzer. This research was carried out at the Department of Ceramics and Refractory Materials, Faculty of Materials Science and Ceramics, AGH University of Krakow. Before the test, the samples were sprayed with coal. ImageJ software was used to analyze the microstructure. The scaffold’s porosity, size, and geometry of the pores were estimated. Scaffold porosity (*P*_s_) was determined by
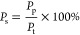
1in which *P*_p_ is the pore surface area and *P*_t_ is the analyzed image area. The average pore size *D* and the aspect ratio *K* (defined as the ratio of the length of the major pore axis to that of the minor) were, respectively, calculated by
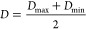
2
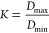
3in which *D*_max_ is the largest and *D*_min_ is the shortest diameter of each pore.

### 2.4 Permeability Evaluation

Permeability parameters were determined using experimental data and fitting of Forchheimer’s eq (eq 4), a well-established empirical relationship that expresses the parabolic dependence of pressure drop (Δ*P*) with the resulting superficial velocity (*v*_s_) of the fluid through the medium.^22−26^
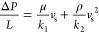
4in which *L* denotes the medium length or thickness along the macroscopic flow direction and μ and ρ denote the fluid’s viscosity and density, respectively. The parameters *k*_1_ and *k*_2_ are referred to as Darcian and non-Darcian permeability coefficients, respectively. These coefficients are dependent only on the porous structure and are used in eq 4 to balance the effects of viscous and inertial losses on the total pressure drop. For compressible flow, Δ*P* must be determined by
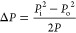
5in which *P*_i_ and *P*_o_ are the inlet and outlet absolute gas pressures, respectively. *P* denotes the absolute pressure at which *v*_s_, μ, and ρ are measured or calculated (in this work *P* = *P*_o_).

Experimental evaluation of permeability coefficients *k*_1_ and *k*_2_ was carried out in a laboratory-made apparatus, with tests performed in a steady-state regime with dry airflow at room conditions (*T* = 26 °C, *P*_atm_ = 94.7 kPa, μ = 1.86 × 10^–5^ Pa·s; ρ = 1.12 kg/m^3^) on 3 specimens of each composition. The cubic sample (≈12 mm × 12 mm × 12 mm) was laterally sealed within a chamber that provided a circular flow area (*A*_flow_) of 16.6 mm^2^, for a useful medium diameter of 4.6 mm. The pressure drop across the specimen (*P*_i_ – *P*_o_) was measured by two digital micromanometers (Dwyer Mark III, model 475, MI) in response to variations in the air volumetric flow rate *Q*, controlled by a needle valve, and measured with a rotameter (Conaut, São Paulo, Brazil) open to the atmosphere. Flow rate (*Q*_N_) was corrected to the value at the sample exit (*Q*_o_) and finally converted to superficial velocity by *v*_s_ = *Q*_o_/*A*_flow_. The setup is schematized in Figure S1. Further details of the method and the experimental setup are given elsewhere.^22−26^

### 2.5 Mechanical Characterization

Mechanical properties were measured in a compressive test using a universal testing machine (Zwick 1435). The compression speed was 5 mm/min. The test was finished when the displacement reached 50%. Young’s modulus (E) was calculated in the range of compression stress 3–5 N. Compression stress (σ) was calculated for 6 and 50% of scaffold deformation (ε). The statistical analysis was performed with a Student’s *t* test (with a confidence level of 0.95). The data were expressed as the mean ± the standard deviation.

### 2.6 Statistical Analysis

Every data point was obtained from three parallel samples in every group of materials. All data were calculated using the mean ± the standard deviation (SD). Every data in microstructural analysis were obtained from a minimum of 100 pores from each sample.

## 3 Results and Discussion

### 3.1 Microstructure

**Figure 2:**
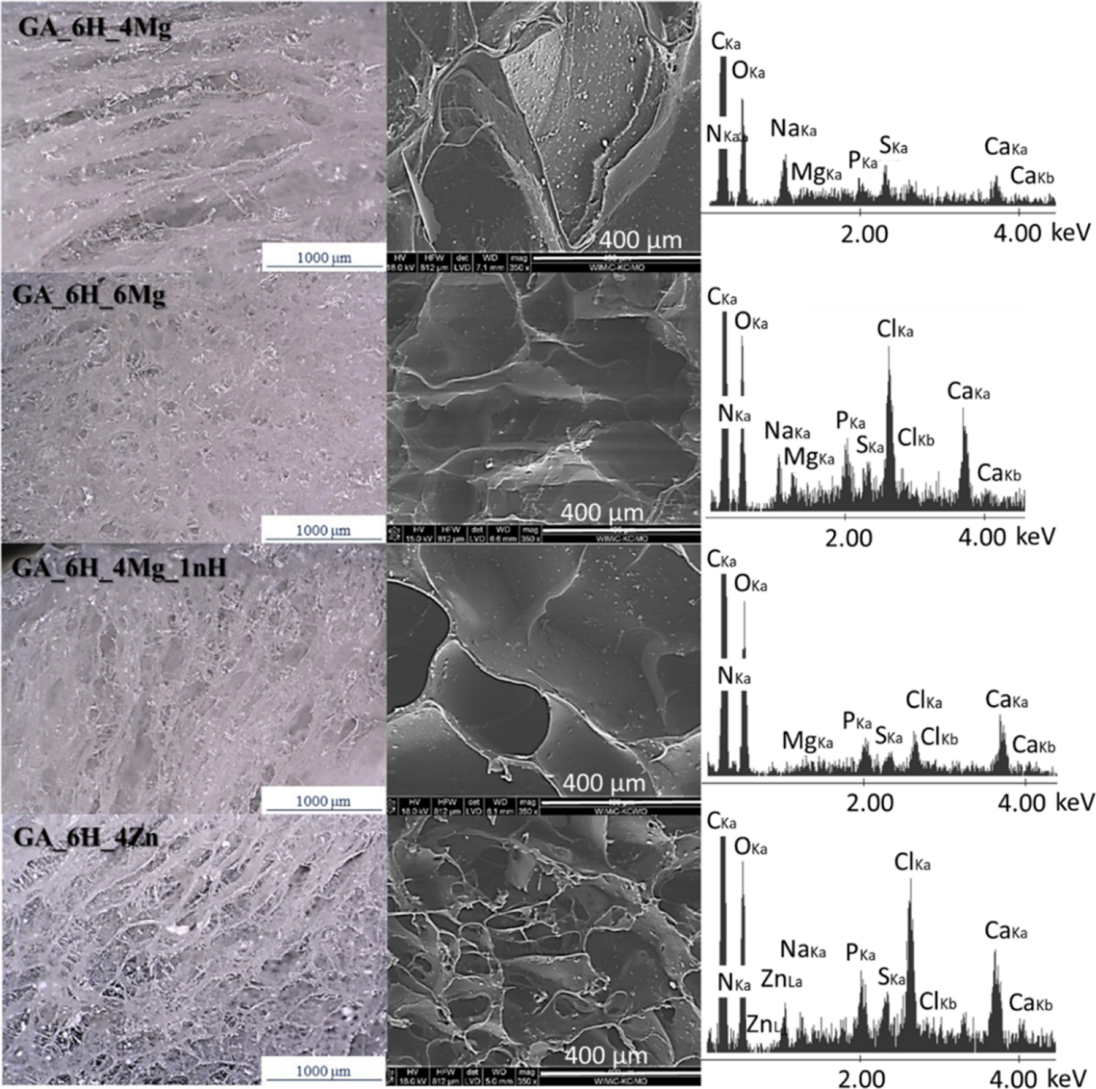
Microstructure of samples obtained by optical microscope and SEM with average EDS analysis (cross section in the plane of “*yz*” according to the axes in Figure 1).

The microstructure and elemental analyses of scaffolds are given in Figure 2. The EDS results (average for the image) indicated the presence of Ca, P, Zn, and Mg. Due to the minute amount of Mg added to the composites, the Mg signal was quite weak. The average content of Mg measured in EDS was 0.32 ± 0.04 wt % for samples with 1.9 wt % of MgCl_2_ and 0.52 ± 0.04 wt % for sample with 2.9 wt % of MgCl_2_. The average content of Zn was 3.94 ± 0.05 (GA_6H_4Zn), P was 3.21 ± 0.14, and Ca was 5.57 ± 0.17. Calcium comes from HAp and alginate.

The scaffolds presented a porosity gradient depending on freeze-drying process, especially freezing parameters, container shape, and heat transfer coefficient to form (Table 2). The increase of the porosity and the pore size was observed from the surface of the samples, which did not come into contact with the form (sample’s skin), through the intermediate area to its interior (middle part). Porosity was greatly reduced at the edges of the sample, which were in contact with the bottom of the rounded mold, as schematized in Figure 1 (indicated by arrows). Solvent freezing kinetics produced such a microarchitecture. Crystals in the middle of the samples had more time to grow. Furthermore, the silicon container had a low heat transfer coefficient, which slowed the rate of crystallization in relation to the mold’s top. Tran et al.^27^ described a similar gradient microstructure obtained in freeze-drying methods. The gradient microarchitecture was obtained by dipping the precooled Teflon rod into the poly(l-lactide-*co*-e-caprolactone) solution, forcing an immediate freeze on the surface, followed by a gradual freezing of the solution on the top of the initial frozen layer.

**Table 2:** Porosity of the Gelatin/Alginate Composites

	porosity [%]		
	near-surface area (sample’s skin)	intermediate area	middle area
GA_6H_4Mg	29 ± 6	41 ± 9	74 ± 9
GA_6H_6Mg	15 ± 7	53 ± 7	67 ± 8
GA_6H_4Mg_1nH	25 ± 7	44 ± 3	70 ± 8
GA_6H_4ZnO	28 ± 6	52 ± 8	75 ± 8

Additionally, distinguishing characteristics of the pores can be observed in Figures 1 and 2. At the top of the sample (from the side in contact with the surrounding environment during freezing), the pores were smaller, irregularly shaped, and dispersed in random directions. In the middle of the samples, the pores were parallel and elongated in the “*z*” direction. The aspect ratio *K* (defined as the ratio of the length of the major pore axis to that of the minor) reached a value of 9. As stated previously, such gradient scaffolds are promising materials for osteochondral defects,^6^ due to microstructural, mechanical, and biological differences between cartilage and subchondral bone.

Considering the influence of modifiers on the porosity of samples (Table 1), the observed differences are not statistically important. However, there was a tendency (mainly in the near-surface and middle part) of slightly higher porosity in the case of GA_6H_4Zn and GA_6H_4Mg samples. In the literature, the influence of additives on the scaffold’s porosity was different depending on their type and content as well as the type of matrix and interaction at the interfaces. Very often, the porosity decreased with increasing in the share of modifier.^20^ However, in the other works, the effect of additives on porosity was not observed.^20,28,29^ The influence of such modifiers on sample porosity should be considered because of their differing effects on the crystallization process of water during solution freezing. The kinetics of nucleation and growth of water crystals vary depending on particle size, contribution, and dispersion.^20,28,29^ Porosity is created by the sublimation of water during the freeze-drying process, and it replicates the microarchitecture of water crystals in a polymeric network.

**Figure 3:**
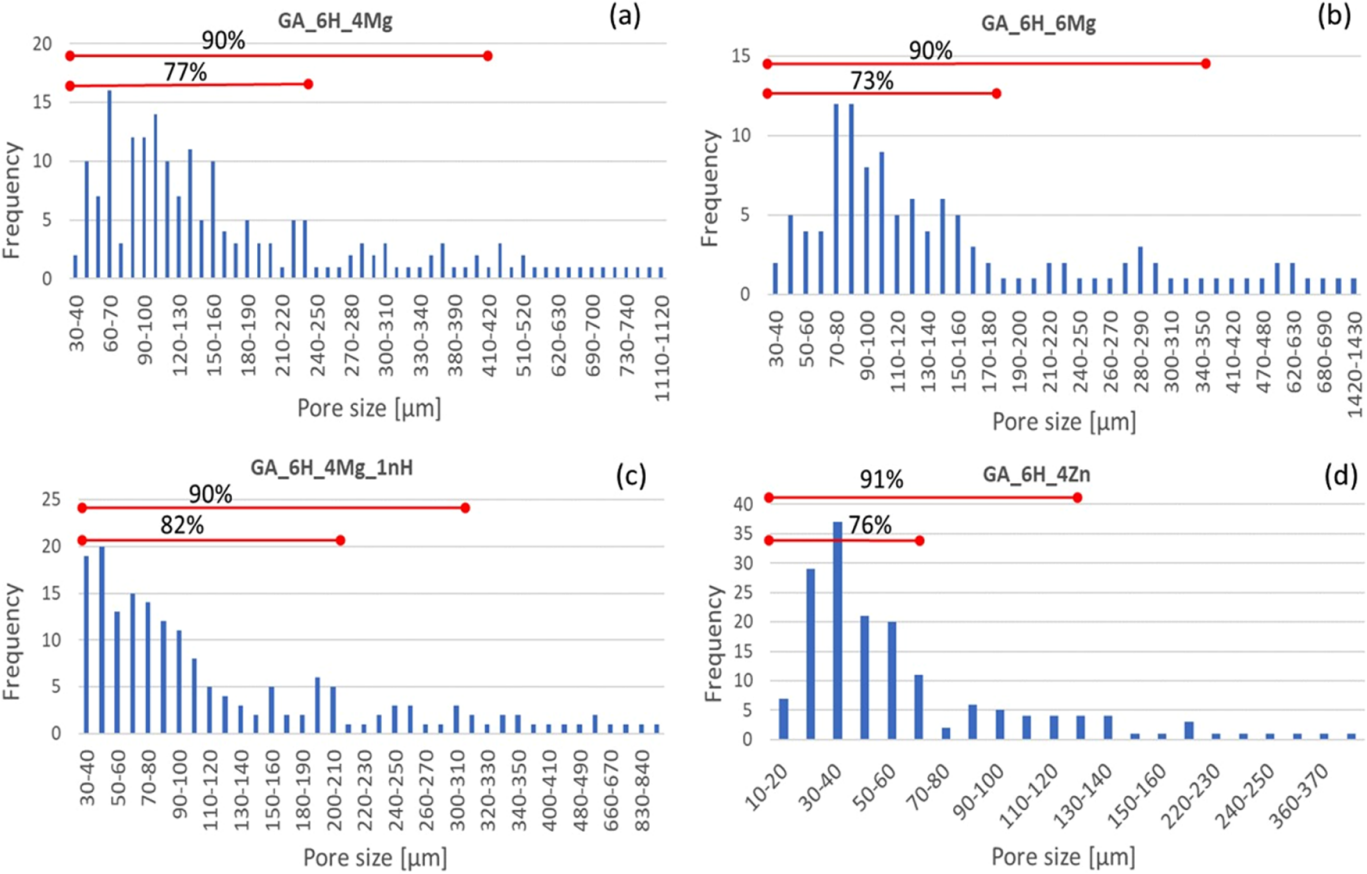
Distribution of the pore size for scaffolds (in the *z* direction of samples): (a) GA_6H_4Mg, (b) GA_6H_6Mg (b), (c) GA_6H_4Mg_1nH, and (d) GA_6H_4Zn.

The pore size distributions of the four scaffold formulations are listed in Figure 3.

The size distributions for samples differing in MgCl_2_ content were quite similar, as observed in Figure 3a,b. In the case of the sample GA_6H_4Mg, about 90% of the pores had a diameter in the range 30–420 μm, with a maximum pore diameter of 1120 μm. For the sample GA_6H_6Mg, about 90% of the pores had a diameter in the slightly narrower range of 30–350 μm, even though larger pores appeared (up to 1430 μm). The presence of such large pores may have resulted from the introduction into the solution of an additional amount of water from the applied hydrate (MgCl_2_·6H_2_O). The presence of structural water connected with additives may have facilitated an easier concentration of water during freezing and ice crystallization.

The greatest impact on the pore size was recorded for scaffolds containing ZnO (Figure 3d). More than 90% of the pores were in the range of 10–130 μm, 76% of the pores were in the very narrow range of 10–70 μm, and the largest pore reached a diameter of 420 μm. As described previously, this additive had little effect on the porosity of the substrates. These results suggest that the kinetics of ice crystal formation differ. Probably, the particles of the modifier were the germs of crystallization and rapid nucleation of a significant number of crystals occurred. The formed crystals exhibited limited growth, most likely as a result of the highest total content of modifiers; there was also the highest number of forming crystals, which may have inhibited the reorganization of hydrogel molecules, the higher concentration and regular arrangement of water molecules, and the growth of ice crystals. The formation of thinner ice crystals reduces the pore size of scaffolds.^20,30^ The ZnO particles were in submicrometer size, which results in their larger number in a given sample volume and thus a larger number of crystallization germs.

A clear difference in pore size could be observed after the introduction of 1 wt % nanohydroxyapatite (Figure 3c), but it was not as significant as in the case of the ZnO additive. In the presence of a HAp nanoadditive, the pores were clearly smaller in diameter than in the case of an analogous sample without a nano-HAp (comparing GA_6H_4Mg_1nH and GA_6H_4Mg). 90% of the pores were in the range of 30–310 μm and, the maximum pore size was 930 μm. It can be assumed that the presence of nano-HAp promoted nucleation and uniform growth of crystals during freezing. A similar relationship regarding the effect of HAp on the porosity and pore size of scaffolds obtained by freeze-drying can be found in the literature.^20^ Ufere et al. achieved the average pore size of 124 μm for polycaprolacton (PCL) scaffolds and 92 μm for polycaprolactone/Hap scaffolds.^28^ Choi et al. obtained the similar results observing a decrease in pore size with an increase in the share of HAp in the PCL composite.^29^ At the same time, they did not register a change in the porosity.

Analyzing the factors affecting the process of solvent crystallization in obtaining scaffolds with freeze-drying method, one can distinguish, the share of modifiers, their size and properties (e.g., conductive properties), the concentration of the solution (including its viscosity), and the temperature and rate of cooling.^30,31^ Taking into account the ceramic modifiers, the most commonly described relationship was the reduction of the average pore size with an increase in the share of additives, which, in some studies, was also associated with a decrease in the porosity of scaffolds.^20^ This may explain the greatest impact of ZnO on the reduction of pore size, the share of which was 4 wt %, while the share of MgCl_2_ was 1.90 and 2.88 wt %, respectively. The two-stage freezing process used in the work, initially at −20 °C, heating to approximately 0 °C, and refreezing at −80 °C, allowed us to obtain considerable pore sizes despite the addition of modifiers. This approach is known in the literature, and Fereshteh described the exact kinetics of ice crystal formation depending on the freezing parameters.^30^

Except for nano-HAp, all samples presented a normal distribution of pore size with the highest size frequency in the range of 73–76%. The remaining pores of a larger dimension occurred individually. On the other hand, the pore size distribution for the nano-HAp sample was asymmetrical, due to the presence of numerous pores in the lowest measuring ranges. This can be related to the nanometric size of the particles, favoring the formation of a higher number of smaller ice crystals in the freezing process. Such a hierarchical microstructure of scaffolds is desirable in the regeneration of osteocartilage defects because it enables neovascularization and cell’s migration.^32^ Loh and Choong described in the literature review, that the minimal pore size necessary for the angiogenesis was approximately 30–40 μm to enable the exchange of metabolic components and to facilitate endothelial cell admission.^32^ However, the larger pore sizes of approximately 160–270 μm facilitated the regeneration of blood vessels. A similar effect was observed with osteoblasts. Smaller pores with a diameter of ∼40 μm, due to a greater surface development, promoted the osteoblast attachment, while pores larger than 100 μm facilitated cell migration. The pores greater than 300 μm had a beneficial effect for cell proliferation and infiltration. There was observed for chondrocytes. Summarizing, the hierarchical porosity of scaffolds promoted the processes of angiogenesis, osteogenesis, and chondrogenesis.

### 3.2 Permeability

The permeability analysis of porous materials is important not only to allow the prediction of the action-response (i.e., pressure–flow rate) for a given fluid flow conditions but also to investigate and correlate the quality of the pores with the processing conditions. Three cubic samples (∼12 mm) of scaffolds derived from the four formulations were subjected to an assessment of their permeability to airflow. The length-normalized pressure drop curves (Δ*P*/*L*) obtained in the airflow permeation tests for scaffold samples with different modifiers are given in Figure S2. Flow direction was in the *x*-axis, as previously shown in Figure S1.

Forchheimer’s equation [eq 1] was suitably fitted to experimental data (*R*^2^ > 0.99 in all cases), indicating the contributions of both viscous-linear [μ*v*_s_/*k*_1_] and inertial-quadratic [ρ*v*_s_^2^/*k*_2_] terms on pressure drop. A relatively wide dispersion in the slopes of the 3 curves for each material, indicating a variable permeation level. The sample containing zinc (GA_6H_4Zn) was the most permeable, presenting the lowest pressure drop level for 2 of the 3 tested samples (Figure S2d).

**Figure 4:**
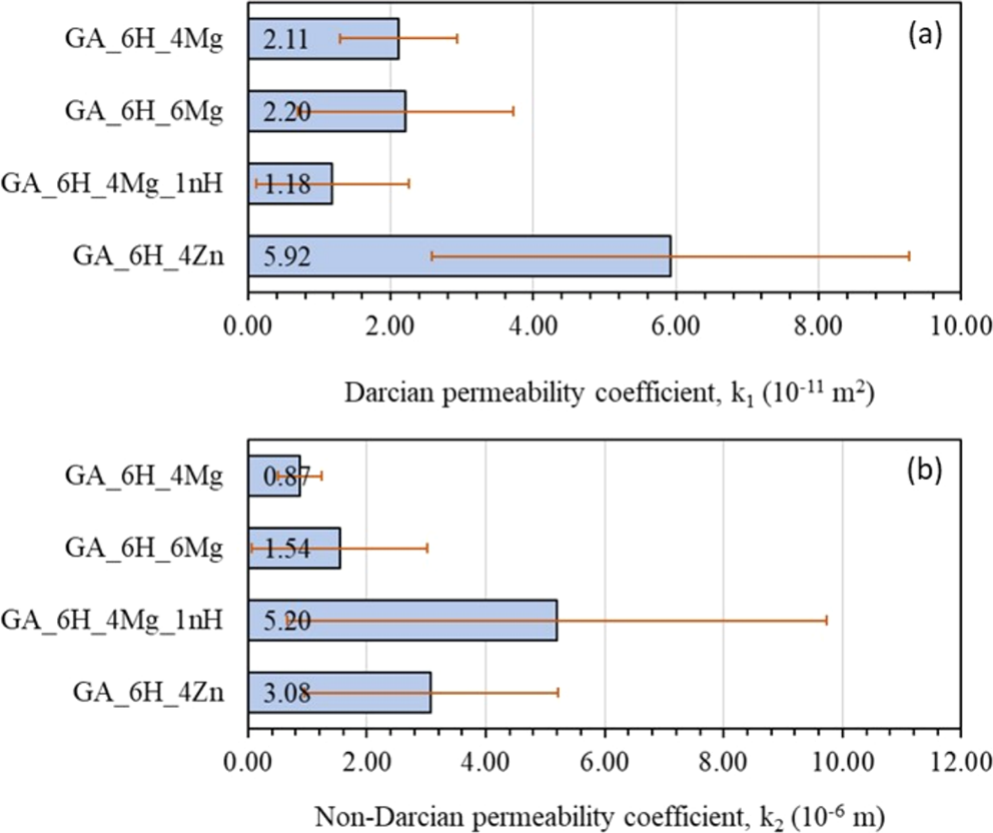
Permeability parameters of scaffolds with different modifiers: (a) Darcian coefficient *k*_1_ and (b) non-Darcian coefficient *k*_2_.

The permeability coefficients (*k*_1_ and *k*_2_) retrieved from the experimental data are presented in Figure 4.

There was no statistically significant impact of the magnesium content on the permeability of the scaffolds. This is evident from the similar average values of the Darcian coefficient (*k*_1_) observed in samples GA_6H_4Mg and GA_6H_6Mg. This discovery provides further support for the observed patterns in the pore size distribution, which exhibited similarities in both scaffolds containing magnesium. The sample containing nanohydroxyapatite (GA_6H_4Mg_1nH) exhibited the lowest mean *k*_1_ value of 1.18 × 10^–11^ m^2^, whereas the sample containing zinc (GA_6H_4ZnO) displayed the highest *k*_1_ values of 3.08 × 10^–11^ m^2^, albeit with a wider range of variation. Comparable levels of variability were noted for the non-Darcian coefficient *k*_2_, with the exception of the sample containing nanohydroxyapatite, which exhibited the greatest degree of variation.

The values of *k*_1_ and *k*_2_ are expected to exhibit a direct relationship with the porosity and average pore size of the structure, as indicated by various empirical relationships documented in the existing literature.^27^ However, the results of this study suggest that the relationship among porosity, pore size, and permeability was inconclusive. Specifically, the data presented in Table 2 for porosity, Figure 3 for pore size, and Figure 4 for permeability do not provide conclusive correlation evidence. This suggests that the interconnectivity of pores may have had a significant influence on the permeability of scaffolds with varying modifiers. The freeze-drying method is suitable for obtaining scaffolds with pore interconnectivity because such a microstructure is naturally created during solid solvent evaporation.

Although the laboratory experiments in this study were performed under conditions involving airflow, it is important to note that scaffolds in real-world scenarios are likely to come into contact with various liquids, such as blood and other bodily fluids. Permeability is a crucial parameter that plays a significant role in characterizing the scaffold’s ability to facilitate extracellular matrix (ECM) infiltration, nutrient exchange, metabolic waste removal, and subsequent cell migration and proliferation. The assessment of the relative importance of *k*_1_ and *k*_2_ in different fluid flow scenarios can be conducted by utilizing the dimensionless parameter (Fo), commonly known as Forchheimer’s number. This parameter is defined as follows
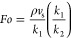
6Based on the *Fo* parameter, eq 4 can be rewritten as
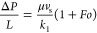
7The parameter *Fo* is analogous to the Reynolds number (*Re*) in its association with the linearity of the pressure drop curve, just as *Re* is commonly associated with the laminar flow in ducts. Upon examination of eqs 4, 6, and 7, it can be discerned that when the Fourier number (*Fo*) is significantly smaller than 1, only the term [μ*v*_s_/*k*_1_] holds significance in predicting the ratio of pressure drop (Δ*P*) to length (*L*), as indicated by Darcy’s law. Conversely, in cases where *Fo* ≫ 1, the change in pressure per unit length (Δ*P*/*L*) can be reasonably estimated using the expression [ρ*v*_s_^2^/*k*_2_]. To accurately assess the overall Δ*P*/*L* under conditions of intermediate flow, it is essential to employ the comprehensive Forchheimer’s equation, which encompasses both coefficients *k*_1_ and *k*_2_. The percentage contributions of viscous (Δ*P*_viscous_) and inertial (Δ*P*_inertial_) pressure drops can be easily computed from
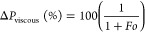
8
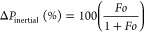
9The value of *Fo* is dependent on the fluid properties, specifically viscosity (μ) and density (ρ). Consequently, the relative contributions of viscous and inertial terms may vary significantly for a given porous sample, depending on whether the fluid is a gas or a liquid. In this study, it was observed that the maximum airflow velocity remained nearly constant at 2.0 m/s across all of the experimental tests. However, the resulting Fo values exhibited significant variation, ranging from 0.09 to 4.05, depending on the specific sample being examined. As a result, the influence of inertia was found to be substantial (with a contribution of 8–80% for Δ*P*_inertial_ and 92–20% for Δ*P*_viscous_), thereby rendering Darcy’s law inadequate for predicting the correlation between Δ*P* and *v*_s_ in the context of airflow.

A simulation was subsequently conducted using eqs 4 and 6–9 to compare the pressure-flow response of each scaffold generated in this study, utilizing the specific *k*_1_ and *k*_2_ values depicted in Figure 4 and the water properties at 25 °C (μ = 8.94 × 10^–4^ Pa·s and ρ = 996.7 kg/m^3^). A fixed scaffold thickness of *L* = 1 cm was adopted, with a fixed water velocity *v*_s_ = 1 cm/s. The results of the simulation are listed in Table 3.

**Table 3:** Simulation Results for the Permeation of Water through the Scaffolds with Different Modifiers

sample	*Fo*	Δ*P*_total_ (Pa)	Δ*P*_viscous_ (%)	Δ*P*_inertial_ (%)
GA_6H_4Mg	0.27	5385	78.7	21.3
GA_6H_6Mg	0.16	4708	86.2	13.8
GA_6H_4Mg_1nH	0.03	7737	97.5	2.5
GA_6H_4Zn	0.21	1833	82.3	17.7

The data presented in Table 3 confirm the previous observation that nanohydroxyapatite yielded the scaffold with the lowest permeability, whereas zinc yielded the most permeable scaffold. Table 3 also shows that, with the exception of the scaffold based on nanohydroxyapatite, the impact of inertia on the pressure drop was significant (ranging from 17.7 to 21.3%), even at a relatively low water velocity of 1 cm/s. Blood velocities in human vessels can reach up to 100 cm/s, resulting in the potential for significantly increased inertial effects. The primary implication of this finding is that the estimation of pressure drop in scaffolds under realistic circumstances should not rely on Darcy’s law. Therefore, it is necessary to determine both *k*_1_ and *k*_2_ through laboratory experiments rather than solely relying on the Darcian coefficient *k*_1_ as commonly reported in the literature. Syahrom et al.^33^ presented a comprehensive review of only Darcian permeability (*k*_1_) data for different types of natural and synthetic cancellous bone structures. Innocentini et al.,^22^ on the other hand, included values for both *k*_1_ and *k*_2_ in a comparison with other scaffold and natural bone structures. Values of permeability of PCL scaffolds were observed in a broad range depending with manufacturing method and scaffold porosity, and they are within the range of 1.42 × 10^–13^ to 15.37 × 10^–10^ m^2^.^3,34,35^^3,34,35^ However, for natural bone the values of permeability are also changed in a broad range depending on the bone type and flow direction (from 1.2 × 10^–10^ m^2^ for human proximal femur in transverse direction to 743 × 10^–10^ m^2^ for human lumbar vertebrae in superior-inferior direction).^33,36^ According to Nauman et al., the intertrabecular permeability ranged from 2.68 × 10^–11^ to 2.00 × 10^–8^ m^2^.^36^ The aforementioned attribute of the bone arises from its gradient structure and the influence of external forces on the bone trabeculae, as described by Wolff’s law.

In the case of hydrogel scaffolds, their parameters such as mechanical properties and permeability depends also on the presence of micropores, hydrophilicity, and cross-linking degree.^37^ Jeong and Hollister obtained 3D poly(1,8-octanediol-co-citrate) (POC) scaffolds with and without hydrogel (collagen I gel) for cartilage regeneration and with porosity 32, 44, and 62%.^38^ They described that permeability decreased significantly from 5.24 × 10^–9^ m^2^ for scaffold without gel to 0.41 × 10^–9^ m^2^ for scaffold with gel. Furthermore, it was observed that the regression coefficients exhibited no significant dependence on porosity in scaffolds containing a gel. Conversely, in the case of scaffolds lacking a gel, a linear correlation was observed between permeability and porosity. Authors attribute this phenomenon to a more intricate correlation between the permeability and the architecture of scaffolds. It can be inferred that water has the potential to permeate the hydrogel structure due to its swelling behavior, which subsequently alters the porosity of the hydrogel. The degradation time of the scaffold with the gel was found to be nonlinearly dependent on porosity, which is an important factor to consider. The researchers observed that the 62% porous scaffolds exhibited the highest degradation rate and the fastest degradation over time. It is noteworthy that the pore structure remains unaltered after a period of 3 weeks of degradation, in contrast to scaffolds possessing lower levels of porosity. The results presented in this study demonstrated the intricate characteristics of hydrogels. Based on the available evidence, it can be inferred that hydrogels possess the ability to facilitate cell proliferation despite their initially low permeability values. This can be attributed to the significant swelling and subsequent increase in scaffold permeability, as the hydrogel degrades over time.

**Figure 5:**
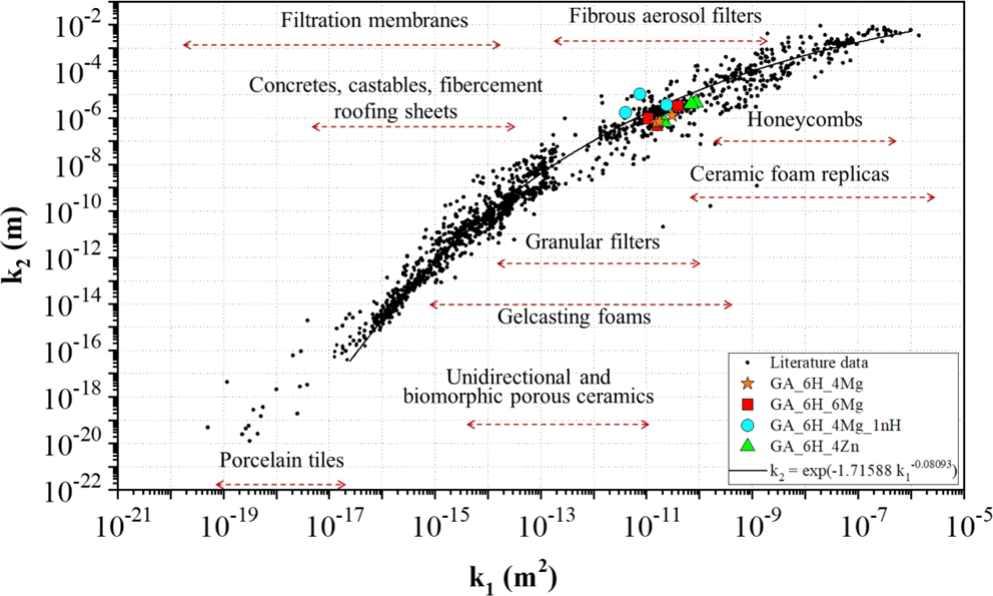
Permeability map with the location of scaffolds produced with different modifiers in this work. Adapted from the works of Innocentini et al.^22−26^

The scaffolds generated in this investigation can also be evaluated alongside other porous materials using the permeability map depicted in Figure 5, based on the works presented in the literature.^22−26^ The inclusion of modifiers led to the formation of scaffolds that exhibited permeation levels (ranging from 10^–12^ to 10^–10^ m^2^ for *k*_1_ and from 10^–5^ to 10^–7^ m for *k*_2_), which were comparable to those observed in gelcasting foams, biomorphic ceramics, and granular filters.

### 3.3 Mechanical Properties

**Figure 6:**
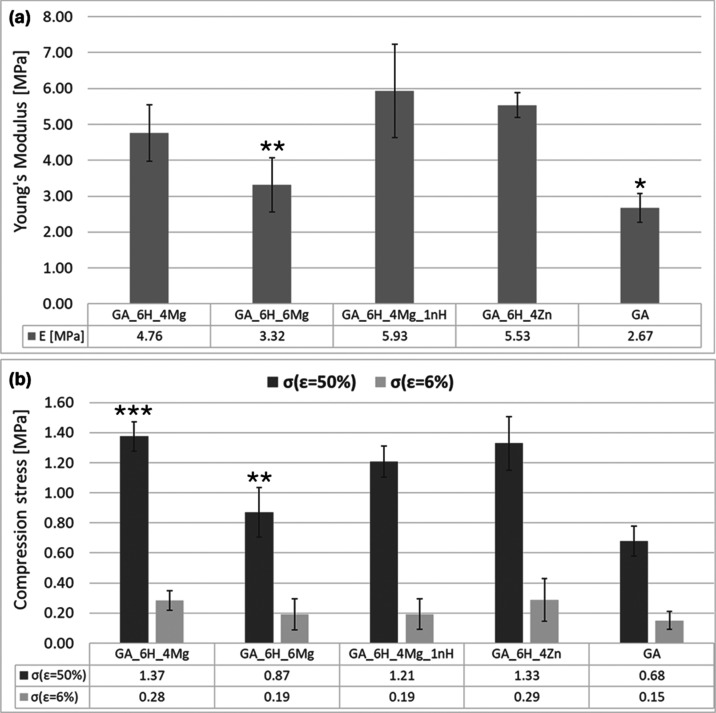
Mechanical properties of scaffold samples measured in the *z* direction: (a) Young’s modulus and (b) compression strength. The results are shown as means ± SD. (*) Statistically significant GA compared to GA_6H_4Mg, GA_6H_4Mg_1nH, and GA_6H_4Zn (*p* < 0.05). (**) Sstatistically significant GA_6H_6Mg compared to GA_6H_4Mg_1nH and GA_6H_4Zn (*p* < 0.05). (***) Statistically significant GA_6H_4Mg compared to GA_6H_6Mg and GA (*p* < 0.05).

All modifiers improved the mechanical properties of the scaffolds. Young’s modulus was 2.7 MPa for the pure scaffold gelatin/alginate without any modifiers, and compression stress (σ_ε = 50%_) was approximately 0.7 MPa (Figure 6). According to the literature, such mechanical parameters are satisfied for pure hydrogels and are obtained by combining gelatin and alginate to form an interpenetrated polymer network.^21^ Wen et al. increased the compressive strength of gelatin from 0.16 to 1.69 MPa by increasing the alginate concentration in gelatin from 0 to 3% w/v.^39^ Except for GA_6H_6Mg, all composites achieved the highest mechanical parameters (*E*, σ) with differences within the limits of statistical error.

Mechanical studies have shown a significant effect of the contribution of the additives. Increasing the quantity of MgCl_2_ from 1.9 to 2.9 wt % caused a decrease in Young’s modulus and a significant decrease in compression stress. This indicates the importance of the content of this modifier, and it is probably related to the pore size distribution. As was mentioned above, the individual pores in scaffold with 2.9 wt % of MgCl_2_ (GA_6H_6Mg) had a large size reaching even 1430 μm. The presence of such heterogeneities may result in significant deterioration of the mechanical parameters of the scaffolds. For scaffold GA_6H_4Mg (with smaller content of MgCl_2_), the mechanical parameters are significantly higher.

A significant improvement in mechanical parameters was observed with the incorporation of 4 wt % of ZnO. This phenomenon can be attributed to the increased uniformity of pore size, which is characterized by a reduced dispersion in their dimensions as well as the existence of pores with a smaller diameter. It is widely recognized that the incorporation of ceramic additives into a polymer matrix leads to enhanced strength, in accordance with the law of mixtures.^40^ The reinforcement effect is intricately linked to various factors, including the nature, concentration, and dimensions of the particles as well as the characteristics of the interfaces (such as the potential interactions between the polymer and modifier, which are contingent upon the specific polymer type). The mechanical parameters may deteriorate when the share of the modifier exceeds a critical value, as a result of the composite’s inferior homogeneity. The mechanical test results demonstrate the favorable homogeneity of the GA_6H_4Zn and GA_6H_4Mg scaffolds.

The anticipated strengthening resulting from the incorporation of nanoadditives in the GA_6H_4Mg_1nH scaffold was not realized. The inclusion of 1% nano-HAP resulted in a slight reduction in compression stress when compared with the analogous GA_6H_4Mg sample. However, this observed change does not possess a statistical significance. This observation was made despite the fact that the scaffold with nano-HAp had a smaller pore size. It is widely recognized that nanoadditives typically enhance the mechanical properties of composites to a limited extent. However, their propensity for agglomeration is attributed to their significant surface area expansion.^41^ Hence, the achievement of a uniform dispersion of the nanoadditive within scaffold GA_6H_4Mg_1nH may have been challenging due to the concurrent presence of additional additives, namely, 6 wt % of HAp microparticles and 1.9 wt % of MgCl_2_. The interface compatibility of hydroxyapatite particles with the matrix may affect their homogeneity. The researchers Tomić et al.^19^ conducted a study to investigate the impact of hydroxyapatite on the Young’s modulus and porosity of gelatin and gelatin/alginate scaffolds. An increase in Young’s modulus was observed following the modification of gelatin with 5 wt % HAp, resulting in a change from 2.08 to 2.76 MPa. This increase was observed independently of any effect on the porosity. Nevertheless, a distinct outcome was achieved through the alteration of the gelatin/alginate matrix, resulting in a decrease in both porosity and Young’s modulus. The authors established a correlation between this phenomenon and the agglomeration process, resulting from the inadequate interfacial compatibility between HAp particles and the alginate matrix. It is imperative to identify an additive that can strike a balance between enhancing the mechanical properties and maintaining a high level of porosity in the scaffolds. This work successfully achieved a compromise for the proposed scaffolds. Notwithstanding the variations among the specific composites, the mechanical parameters obtained are deemed appropriate for their utilization in the field of cartilage tissue engineering.

According to the literature, this type of scaffold has a compressive strength of 0.01–3 MPa.^42^ Haung et al. optimized the biomechanics of cartilage growth through the application of varying pressures of 0.04–0.34 MPa on gelatin and alginate scaffolds.^42^ Because the compressive modulus of cartilage varies from 0.2 to 6.44 MPa depending on the zone,^6^ the Young’s modulus achieved for the obtained scaffold is also satisfactory and should not disrupt cartilage biomechanics.

## 4 Conclusions

The freeze-casting technique used in this work allowed the processing of porosity-gradient scaffolds. The central region of the samples had the maximum porosity (67–75%) and the largest and elongated pores. The microarchitecture of the pores was due to the water crystallization process during solution freezing and was dependent on the specificity of the modifiers. Zinc-containing scaffolds showed the highest permeability despite their modest pore size, indicating strong pore interconnection. The high mechanical characteristics of these samples also suggest a homogeneous distribution of ZnO and pores. Magnesium-containing scaffolds had significantly larger pore sizes (up to 1430 μm); however, this did not result in an increase in permeability but instead caused a decrease in mechanical strength. Finding a correlation between microstructure and permeability in samples containing nanohydroxyapatite proved problematic due to the specificity of the nanoadditive and hydrogels as well as the complexity of their interactions. Nanohydroxyapatite (nHAp) altered the crystallization of smaller ice grains, resulting in the presence of smaller pores (30–310 μm). Additionally, nHAp reduced the strength of the samples, likely due to the presence of agglomerates. Permeability coefficients were used for water flow simulation to mimic body fluids and indicated the significant influence of inertia on pressure drop (excluding nanohydroxyapatite-based samples). This led to a critical conclusion about the need of using coefficients *k*_1_ and *k*_2_ to characterize hydrogel scaffolds for flow of realistic blood-like fluids. Because of their high porosity, suitable pore microarchitecture, excellent permeability, and adequate mechanical properties, the developed composite materials offer potential as biomimetic scaffolds for osteochondral defect regeneration.

## Data Availability

Data will be made available on request.

## Supporting Information Available

Figure S1. Scheme of the permeability apparatus. Figure S2. Pressure drop curves for assessment of air permeability of scaffolds samples in the *x*-direction: (a) GA_6H_4Mg; (b) GA_6H_6Mg; (c) GA_6H_4Mg_1nH; (d) GA_6H_4Zn (PDF)
