# Supplementary material for: Microstructural, Fluid Dynamic, and Mechanical Characterization of Zinc Oxide and Magnesium Chloride-Modified Hydrogel Scaffolds
Source: ACS Biomater Sci Eng. 2024 Jul 16;10(8):4791–801. doi: 10.1021/acsbiomaterials.4c00286 (PMC11322906; doi:10.1021/acsbiomaterials.4c00286)
Supplement: Supplementary file 1 — ab4c00286_si_001.pdf [file ab4c00286_si_001.pdf]

Microstructural, fluid dynamic, and mechanical characterization of zinc oxide and magnesium chloride-modified hydrogel scaffolds

Murilo Daniel de Mello Innocentini [1,2], Bruno Ribeiro Fuzatto Bueno [1], Agnieszka Urbaś [3], Anna Morawska-Chochół [4*]

[1] University of Ribeirão Preto, Course of Chemical Engineering, Av. Costabile Romano 2201, 14096-900 Ribeirão Preto, SP, Brazil

[2] University of Bath, Department of Architecture and Civil Engineering, Centre for Regenerative design and Engineering for a NEt positive World (RENEW), Bath BA2 7AY, UK

[3] AGH University of Krakow, Faculty of Electrical Engineering, Automatics, Computer Science and Biomedical Engineering, Biomedical Engineering, 30-059 Kraków, Poland

[4] AGH University of Krakow, Faculty of Materials Science and Ceramics, Department of Biomaterials and Composites, 30-059 Kraków, Poland

* Corresponding author: morawska@agh.edu.pl, ORCID: 0000-0003-0209-4402

Supplementary materials: 3 pages, 2 Figures.

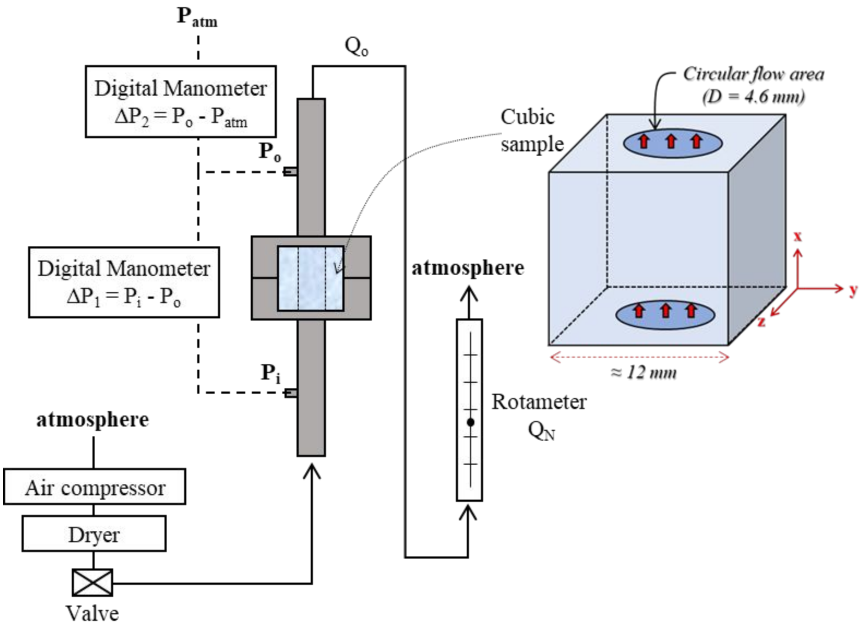

Figure S1. Scheme of the permeability apparatus

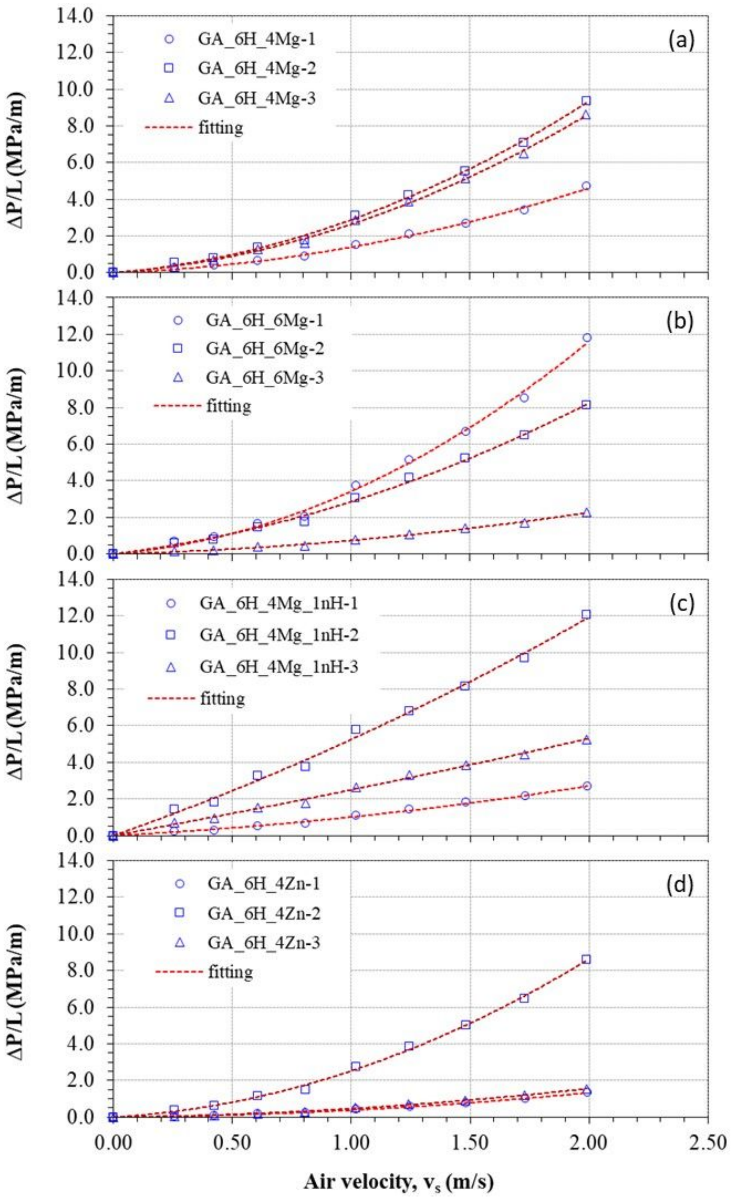

Figure S2. Pressure drop curves for assessment of air permeability of scaffolds samples in the x-direction: (a) GA_6H_4Mg; (a) GA_6H_6Mg; (c) GA_6H_4Mg_1nH; (d) GA_6H_4Zn.
